# Estimation of Iron Availability in Modified Cereal β-Glucan Extracts by an *in vitro* Digestion Model

**DOI:** 10.3389/fnut.2022.879280

**Published:** 2022-06-13

**Authors:** Elena Marasca, Olivia Zehnder-Wyss, Samy Boulos, Laura Nyström

**Affiliations:** Institute of Food, Nutrition and Health, ETH Zürich, Zurich, Switzerland

**Keywords:** iron bioavailability, dialysability, *in vitro* digestion, phytic acid, phytase, β-glucan hydrolysis, sodium periodate oxidation, TEMPO oxidation

## Abstract

For cereal-based foods rich in dietary fibers, iron bioavailability is known to be poor. For native cereal β-glucan extracts, literature has demonstrated that the main factor impacting the bioavailability is phytic acid, which is often found in association with dietary fibers. During food processing, β-glucan can undergo modifications which could potentially affect the equilibrium between phytic acid, fiber, and iron. In this study, an *in vitro* digestion was used to elucidate the iron dialysability, and hence estimate iron availability, in the presence of native, chelating resin (Chelex)-treated, oxidised, or partially hydrolysed oat and barley β-glucan extracts (at 1% actual β-glucan concentration), with or without phytase treatment. It was confirmed that pure, phytic acid-free β-glucan polysaccharide does not impede iron availability in cereal foods, while phytic acid, and to a smaller extent, also proteins, associated to β-glucan can do so. Neither Chelex-treatment nor partial hydrolysis, 2,2,6,6-tetramethylpiperidine 1-oxyl (TEMPO) or NaIO_4_ oxidation significantly influenced the phytic acid content of the β-glucan extracts (ranging 2.0–3.9%; *p* > 0.05). Consequently, as long as intrinsic phytic acid was still present, the β-glucan extracts blocked the iron availability regardless of source (oat, barley) or Chelex-treatment, partial hydrolysis or NaIO_4_-oxidation down to 0–8% (relative to the reference without β-glucan extract). Remarkably, TEMPO-oxidation released around 50% of the sequestered iron despite unchanged phytic acid levels in the modified extract. We propose an iron-mobilising effect of the TEMPO product β-polyglucuronan from insoluble Fe(II)/phytate/protein aggregates to soluble Fe(II)/bile salt units that can cross the dialysis membrane. In addition, Chelex-treatment was identified as prerequisite for phytase to dramatically diminish iron retention of the extract for virtually full availability, with implications for optimal iron bioavailability in cereal foods.

## Introduction

For living organisms, iron is an essential trace element, crucial for biologic functions such as respiration, energy production, DNA synthesis, and cell proliferation (1). Despite its geologic abundance and large-scale intervention programs, more than two billion people in developing as well as high-income countries are affected by iron deficiency, which remains the most widely prevalent nutritional problem worldwide. Besides insufficient dietary intake and pathologic conditions, one of the most likely causes of this nutritional problem is the poor bioavailability of dietary iron, which is influenced by its chemical form, the type of dietary iron (heme or non-heme), the iron status of the individual and the presence of other food components (2–4).

Generally, cereals can be a good source of minerals such as iron and zinc, but their dietary fibers can have a negative effect on their bioavailability (5). Also, there is considerable evidence that phytic acid, which is associated with fibers in many foods, reduces the bioavailability of iron (6–9). In particular, recent studies have confirmed phytic acid as the main responsible for iron-binding by commercial oat and barley β-glucan preparations (10, 11). During food processing, such as baking, sourdough fermentation, heating in aqueous systems, or high-pressure homogenisation, in fact, β-glucan can undergo degradation, e.g., chain scission and oxidation (12–14), which can alter its behaviour in the gastro-intestinal tract, and which can, in turn, affect the equilibrium between phytic acid and iron.

Accurate measurement of iron bioavailability can be challenging, but using iron isotopes in human *in vivo* studies have been proven to be very successful (15). However, since human *in vivo* studies are expensive and time consuming, and animal models have certain limitations, such as the differences in mineral requirements, metabolism, digestive capacity, and sensitivity to dietary factors compared to humans (16), *in vitro* methods for the determination of iron availability on the basis of iron diffusing across a dialysis membrane in a simulated digestion mixture were developed as an alternative in the 1970s and 1980s and are still in use today (17–19). These methods are not suitable to determine bioavailability, since the interactions between the digestive system and the ingested food cannot be measured, and factors such as transit time, site of absorption, composition of digestive secretions responding to the meal and intestinal flora may all affect the mineral availability (19). Despite these limitations, the *in vitro* studies are still very useful to predict trends or compare iron absorption between different samples (15).

While there are several studies concerning iron bioavailability in relation to wheat bran fibers and phytic acid (5, 6, 20), not many studies investigate the impact of other fibers, in particular β-glucan, on the availability of iron. (1→3)(1→4)-β-D-Glucan (cereal β-glucan) is a partially soluble dietary fiber found predominantly in the cell walls of the endosperm and aleurone layer of oat and barley grains and in smaller amounts in rye and wheat. β-Glucan has several positive health effects, in particular blood cholesterol lowering properties, recognised by EFSA as well as the FDA (21, 22), and beneficial blood glucose regulation, as evaluated by EFSA (23).

In a study by Platt and Clydesdale (24), cellulose, lignin, sodium phytate and cereal β-glucan, alone and in combination, were evaluated with regard to their effect on iron profiles in model systems containing FeSO_4_⋅7H_2_O under simulated gastrointestinal pH conditions. The authors observed that lowering the pH to gastric conditions (pH 2) triggered the solubilisation of a portion of the iron bound to lignin, cellulose, and β-glucan. Bringing back the pH to neutral conditions (pH 6.8) led again to increased portions of complexed iron and reduced portions of soluble iron. They hypothesised binding strengths in the order lignin/phytic acid > β-glucan > cellulose, but concluded that the binding components in cereals interact, which critically affects iron solubility and thus bioavailability.

Differences in iron-binding properties of commercial high-viscosity oat and barley β-glucan of high purity (>97%) were also assessed in a previous study (25). The kinetics of the Fenton reaction between iron(II) and hydrogen peroxide in 0.6% oat and barley β-glucan solutions at pH values 2.7 and 4.7 were measured. At low pH, the β-glucan material did not affect the rate of the Fenton reaction, which implies that all iron was available to react with hydrogen peroxide. At pH 4.7, on the other hand, barley β-glucan and even more so oat β-glucan reduced the reaction rate suggesting that both bind iron, with the commercial oat β-glucan material showing a stronger binding power than barley β-glucan. The variation in iron-binding was hypothesised to be caused by the different ratio of β-(1→3)-linked cellotriosyl to cellotetraosyl units (DP3:DP4) between β-glucan from different cereal sources, with oat β-glucan having more β-(1 → 4)-linkages than barley. On the other hand, Fenton-induced oxidation of three constitutionally isomeric β-glucan tetrasaccharides as model compounds with no or one β-(1 → 3)-linkage exhibited no significant differences in degradation kinetics, suggesting that for the commercial β-glucan polymer materials, other constituents than the pure carbohydrates were responsible for the difference in behaviour between the oat and barley β-glucan materials (26). Wang et al. (10, 11) attributed the differences in iron-binding behaviour of commercial β-glucan materials to the varying levels of residual phytic acid, as removal of phytic acid by treatment with ion-exchange resins led to same degradation rates and iron-binding capacities of all β-glucan materials irrespective of their origin.

Thanks to its health promoting properties and technological functions, β-glucan is added as functional ingredient in different food products such as pasta, soups, bread, and low-fat dairy products (27, 28). However, it has been shown that during production and in food systems, β-glucan may be subjected to degradation processes such as oxidation and acid hydrolysis, which alter not only its viscous properties (reduction of *M*_*w*_), but also its interactions with minor food components (13, 27, 29). Literature shows that processing or modification does not necessarily reduce β-glucan’s health benefits or its technological potential, but in certain cases can actually improve both (30–32). However, to be best of our knowledge, no literature investigating the impact of β-glucan structural modifications on iron bioavailability exists. In a previous study (33), we investigated the bile acid retention properties of differently modified β-glucan extracts compared to the native polysaccharide. The modifications included partial hydrolysis and oxidation by sodium periodate and 2,2,6,6-tetramethylpiperidine 1-oxyl (TEMPO). With oxidation and molecular weight reduction not increasing the bile acid retention capability of β-glucan we could show that bile acid retention is purely a function of viscosity. In this study we hypothesise that modification of the cereal β-glucan structure (e.g., oxidatively) changes the iron availability of its extracts. In order to get more insight on how processing affects iron availability, β-glucan extracts from oat bran and barley flour were modified with the different targeted chemical treatments similarly to the aforementioned study and then compared in an *in vitro* digestion experiment. Also, the role of phytic acid was studied by means of prior phytase treatment of the native and chelating resin-treated extracts.

## Materials

Oat flour (OatWell^®^ 28) and barley flour (Beta^®^Barley dehulled and untreated) were supplied by DSM (Herleen, Netherlands) and Dieckmann Cereals GmbH (Rinteln, Germany), respectively. Calcium chloride (CaCl_2_), Chelex^®^ 100 sodium form (50–100 mesh), glycerol, bile extract porcine, concentrated hydrochloric acid (Conc. HCl), iron(II) sulfate heptahydrate (FeSO_4_⋅7 H_2_O), maltotriose, potassium hydroxide (KOH), sodium acetate (NaOAc), sodium azide (NaN_3_), sodium bicarbonate (NaHCO_3_), sodium chlorite (80%; NaClO_2_), sodium hypochlorite solution (available chlorine 10–15%; NaClO), sodium nitrate (NaNO_3_), sodium (meta) periodate (NaIO_4_), 2,2,6,6-tetramethylpiperidine 1-oxyl (TEMPO; 98%) (98%), α-amylase from *Bacillus licheniformis* (Termamyl^®^ 300L), pancreatin from porcine pancreas, and pepsin from porcine gastric mucosa (≥250 U/mg solid) were purchased from Sigma-Aldrich, Switzerland. Ethanol absolute was supplied by Alcosuisse, Switzerland. Acetic acid glacial and sodium hydroxide pellets (NaOH) were purchased from Fisher Chemical, United Kingdom. Sodium hydroxide solution (50–52% in water), sulphuric acid, and sodium dihydrogen phosphate (NaH_2_PO_4_) were purchased from Fluka, Germany. Titrisol^®^ Iron standard (1000 mg in 1000 mL 3.5% HCl) was purchased from Merck, Germany. Ronozyme™ P-(M) (*Peniophora lycii* wt phytase, 10 U/mg), was provided by Novozymes, Denmark. Lichenase (*endo*-1,3:1,4-β-D-glucanase) from *Bacillus subtilis*, 3-*O*-β-cellobiosyl-D-glucose (DP3; >95%), 3-*O*-β-cellotriosyl-D-glucose (DP4; >95%), β-Glucan Assay Kit (Mixed Linkage), and Phytic Acid (Phytate)/Total Phosphorus Kit were obtained from Megazyme, Ireland.

Ultrapure (Milli-Q) water was used for all experiments (Merck Millipore, Merck KGaA, Darmstadt, Germany). All glassware used for the experiments was washed in a laboratory dishwasher, soaked overnight in 1 M HCl and then rinsed with Milli-Q water. All reported contents are given on dry weight basis (dwb) after 110°C drying for 24 h.

## Methods

### β-Glucan Extraction

β-Glucan was extracted and purified from oat and barley flour according to the procedure by Bhatty (34), with slight adaptations. Firstly, the flours were stirred in 1 M NaOH (2% w/w) for 24 h, followed by removal of debris by centrifugation for 10 min at 3220 × *g* and 4°C (Centrifuge 5810 R, Eppendorf, Germany). The supernatant was collected and the pH adjusted to 6.5 with conc. HCl followed by the addition of 2 mL Termamyl (≥300 U/g) and 70 mg CaCl_2_ per liter of extraction mixture. The mixture was then incubated for 1 h at 96°C to hydrolyze starch. After cooling to room temperature (RT), the pH was adjusted to 4.5 with conc. HCl to precipitate proteins, which were removed by centrifugation for 15 min at 3220 × *g* and 4°C. The supernatant was collected and β-glucan precipitated with an equal volume of ethanol. The β-glucan precipitate was collected by centrifugation (10 min, 3220 × *g*, 4°C) and decantation of the supernatant, freeze-dried for 48 h (Lyolab BII, LSL Secfroid, Switzerland), and homogenised with a ball mill (Pulverisette 23, Fritsch, Germany).

### Iron Removal

To remove intrinsic iron from the extracts, a treatment with Chelex (a chelating ion exchange resin) was carried out. Chelex consists of styrene divinylbenzene copolymers containing paired iminodiacetate ions, with a high preference for polyvalent transition metals such as iron. The quantity of ions exchanged is very low at pH below 2, increases sharply from pH 2 to 4, and reaches a maximum above pH 4 (Bio-Rad). Multiple rounds of optimisation led to the following protocol: a 0.75% oat or barley β-glucan solution was prepared by dissolving the extract in Milli-Q water in a shaking water bath overnight (16 h) at 70°C, and subsequently treated three times with Chelex. For each treatment, 50 g Chelex beads were added per liter of β-glucan solution and the suspension stirred for 2 h, then vacuum-filtered through a glass filter (250 mL, pore size 2) (35). After each treatment the Chelex was regenerated with aqueous HCl and NaOH washings as described in the manufacturer’s instructions. In the first and second treatment, the pH of the solution was lowered to 2.5 with 1 and 0.1 M sulfuric acid to favour the solubilisation of iron. For the third treatment, the pH was not adjusted after stirring the solution with Chelex, but left at around pH 10, in order to enhance the chelating power of the resin. After this cycle of treatments, two volumes of ethanol were added to the solutions, which were then stored for 48 h at 4°C to help the precipitation of iron-free β-glucan. The samples were then centrifuged (10 min, 3220 × *g*, 4°C), the pellet freeze-dried for 48 h and milled by ball mill.

### Composition of the Extracts

#### Total β-Glucan Content

The total β-glucan content of the oat and barley β-glucan extracts before and after Chelex treatment was determined in triplicates with the β-Glucan Assay Kit (Mixed Linkage) from Megazyme, Ireland. In this procedure, β-glucan was hydrolysed to β-gluco-oligosaccharides and ultimately to glucose by two incubation steps with lichenase and β-glucosidase, respectively. The amount of liberated glucose was determined by spectrophotometry using a glucose oxidase/peroxidase reagent.

#### Protein Content

To estimate the protein content of the oat and barley β-glucan extracts before and after Chelex treatment, samples were dried for 24 h at 110°C and sent to the service for microelemental analyses at ETH (Elementaranalysen LOC, ETH Zurich). The nitrogen content was determined in quadruplicates by a TruSpec Micro, composed of a combination of flow-through carrier gas and individual, highly selective infrared (IR) and thermal conductivity detectors (TruSpec Micro, LECO Corporation, United States). The approximate protein content was then calculated using the nitrogen conversion factors determined by Mosse (36) of 5.36 for the oat extracts and 5.50 for the barley extracts.

#### Phytic Acid Content

The phytic acid content of the oat and barley β-glucan extracts before and after Chelex treatment was determined in triplicates with the Phytic Acid (Phytate)/Total Phosphorus Kit obtained from Megazyme, Ireland (11). The samples were incubated with phytase and alkaline phosphatase to release inorganic phosphate (P_*i*_) from phytic acid. The total phosphate released was measured with a spectrophotometric method where the amount of formed molybdenum blue is proportional to the amount of P_*i*_ present in the samples.

#### Iron Content

The concentration of accessible intrinsic iron was determined by means of a graphite furnace atomic absorption spectrometry (AAS, Agilent Technologies AG, Switzerland, VARIAN GTA 120) based on the method described by Lynch et al. (37). The wavelength for iron (248.3 nm) was selected, with a slit width of 0.2 nm and a lamp current of 5.0 mA with background correction on. The heating program was divided in several steps to evaporate the water (85–140°C), burn the organic residues (800°C), and atomise the contained iron for the absorption to be measured (2300°C). The calibration curve was automatically constructed by the instrument from a standard solution (10 ng/mL) prepared by dilution of the standard stock solution of Fe Titrisol^®^ (1000 mg/L). After dissolving the β-glucan extracts in water (70°C, 16 h), the solutions were diluted with aqueous HCl to reach a homogeneous solution with a final concentration of 0.1 M HCl (pH 1) and an intrinsic iron concentration below 10 μg/L to lie within the calibration range of the AAS (resulting in 0.01–0.1% final extract concentration depending on the sample). Each sample or standard was measured in triplicate. The samples were stored at 4°C until measurement. Since not a complete digestion of the extracts with concentrated mineral acids was used at high temperatures, the results reflect at the very least the accessible portion of total intrinsic iron relevant for this study, but might potentially be slightly lower than the actual total iron content of the extracts.

### Structural Characterisation of the Extracts

#### DP3 to DP4 Ratio

The fine structure of cereal β-glucan is characterised by the molar DP3 to DP4 ratio, which reflects the relative proportions of cellotriosyl to cellotetraosyl subunits connected by β-(1→3)-linkages along the chain. The DP3/DP4 ratio of oat and barley β-glucan was determined before and after the Chelex-treatment, as well as after the modifications according to a procedure described by Johansson et al. (38). For this purpose, the polymer was hydrolysed by lichenase and the concentration of the resulting main β-gluco-oligomers DP3 and DP4 measured in triplicates with high-performance anion exchange chromatography and pulsed amperometric detection (HPAEC-PAD).

First of all, 70 mg of the different oat and barley β-glucan extracts (untreated, Chelex-treated, hydrolysed, TEMPO- and NaIO_4_-oxidised) were dispersed in 0.2 mL 50% aqueous EtOH and 4 mL 20 mM NaH_2_PO_4_ buffer (pH 6.5), and boiled for 10 min. After cooling, 0.2 mL lichenase solution (20 U/mL) was added and the samples were incubated for 2 h at 60°C. Following inactivation of lichenase by boiling the tubes for 15 min, an aliquot was diluted 33.33-fold and maltotriose added to all samples and calibrants as internal standard (24 μM). Calibration curves were constructed in the range of 10–100 μM for DP3 and 5–50 μM for DP4, relative to the area of the internal standard. The samples, standard solutions for calibration, and water as blank were filtered (0.45 μm) into HPLC vials and analysed by HPAEC-PAD.

The instrument consisted of a gradient pump (GS50), auto sampler (AS50), thermal compartment (AS50), pneumatic controller (PC10) and the electrochemical detector (ED50), all from Dionex Bio LC, United States. The column was an Analytical Dionex CarboPac PA1 (2 mm × 250 mm) with a CarboPac PA1 guard column (2 mm × 50 mm). The signal was measured with pulsed amperometric detection using waveform A from Dionex technical note 21. Eluents were (A) 150 mM NaOH solution and (B) 500 mM NaOAc with 150 mM NaOH. The following eluent gradient was applied: 90% eluent A and 10% eluent B from 0 to 2 min, a linear increase of eluent B from 10 to 100% between 2 and 17 min, 100% eluent B between 17 and 23 min, and 90% eluent A and 10% eluent B from 23 to 37 min. The flow rate was 0.25 mL/min, except between 23 and 36 min, where it was increased to 0.75 mL/min. The resulting peaks were analysed using the software Chromeleon^®^ version 6.8, also by Dionex Bio LC, United States.

#### Molecular Weight

The weight-average molecular weight (*M*_*w*_) of the oat and barley β-glucan extracts before and after the Chelex treatment, as well as after the modifications, was determined in triplicates by means of high-performance size exclusion chromatography (HPSEC) with refractive index detection as previously described (33, 39). The extracts were dissolved in eluent [0.1 M sodium nitrate and 0.02% (w/v) sodium azide] to a final concentration of 0.1% (w/v) and filtered (0.45 μm) into HPLC vials. For the calibration curve, 3 mg of β-glucan standards with reported peak molecular weight (*M*_*p*_) of 33,600, 67,100, 187,100, 247,000, 375,000, and 667,000 g/mol (Megazyme, Ireland) were boiled in 3 mL eluent until dissolved and filtered (0.45 μm) into HPLC vials.

The HPLC consisted of a binary pump, degasser, thermostated column compartment, and auto sampler, all from Agilent Technologies (Series 1100, Hewlett Packard, United States). A pre-column (Viscotek AGuard Col. 50 mm × 6.0 mm, Malvern Instruments Ltd., United Kingdom) was used together with an A5000 column (Viscotek, 300 mm × 7.8 mm, Malvern Instruments Ltd., United Kingdom) and a suprema 30,000 column (10 μm, 8 mm × 300 mm, PSS Polymer Standards Service GmbH, Germany). The temperature of the columns was kept at 35°C with a flow rate of 1 mL/min and an injection volume of 50 μL. The elution was recorded using a refractive index detector [Series 1200, Agilent Technologies (Schweiz) AG, Switzerland]. The weight-average molecular weights were calculated based on the measured standard curve with the ChemStation software (ChemStation for LC 3D systems, Rev B.04.02 SP1) and the add-on Cirrus GPC/SEC software (version 3.4.1) from Agilent.

### Structural Modifications

**FIGURE 1:**
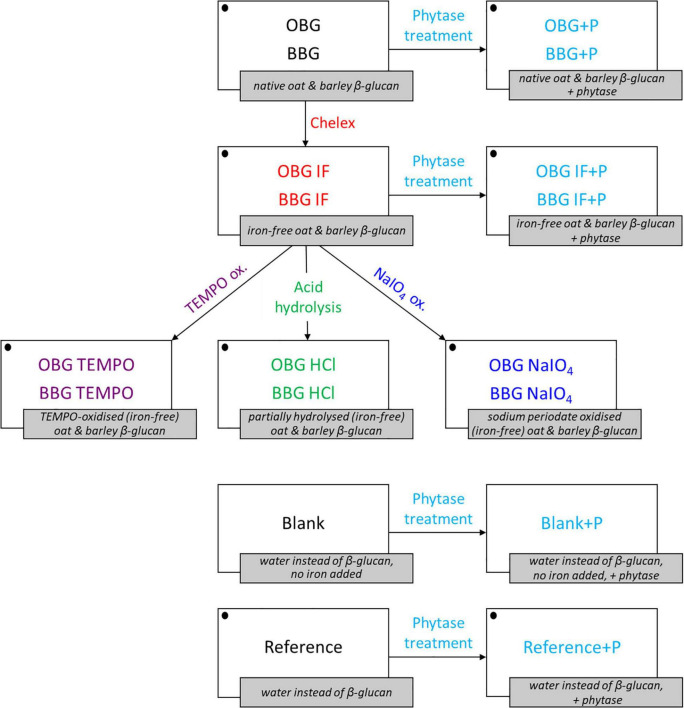
Overview of the β-glucan extract samples, treatments, blanks, and references used for the *in vitro* digestion iron availability experiments. Iron is added for all samples and references (symbolized with • in the figure) with the exception of the blanks (blank and blank+P). OBG, oat β-glucan; BBG, barley β-glucan; IF, “iron-free” (Chelex-treated) samples; +P, phytase treated samples/blanks/references.

The idea behind the extreme modifications performed in this study was to investigate the maximal effect that any processing, such as food production, may have on the iron-binding of the fiber materials. In particular, acid hydrolysis was used to reduce the molecular weight of the fibers without any changes to the β-glucan structure. TEMPO and sodium periodate (NaIO_4_) were used to oxidise the fibers in sort of controlled way, with TEMPO selectively oxidising the primary hydroxyl groups (C6) to carboxylates, and sodium periodate selectively oxidising the two secondary, vicinal hydroxyl groups of the glucose monomers to dialdehydes in the β-glucan molecules (33). An overview of all prepared samples, modifications, and treatments is presented in Figure 1, including blanks, references, and the abbreviations used. Note that all structural modifications (TEMPO oxidation, acid hydrolysis by HCl, and NaIO_4_ oxidation) were done on the Chelex treated, “iron-free” oat and barley samples (OBG IF and BBG IF).

#### Acid Hydrolysis

The pH of 1% (w/v) Chelex-treated oat or barley β-glucan extract solutions (OBG IF or BBG IF) was adjusted to pH 1 with 1 M HCl. The samples were left to hydrolyse for 24 h at 50°C and then neutralised with 1 M NaOH (40). The extracted β-glucan was precipitated by adding two volumes of ethanol and then stored at 4°C for 48 h to help the precipitation. Finally, the samples were centrifuged (10 min, 3220 × *g*, 4°C), the collected solids freeze dried for 48 h (Lyolab BII, LSL Secfroid, Switzerland) and milled by ball mill (Pulverisette 23, Fritsch, Germany).

#### Sodium Periodate Oxidation

**FIGURE 2:**
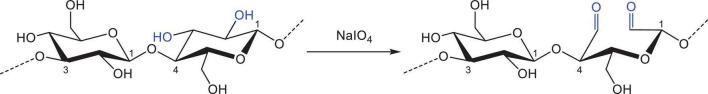
Oxidation of vicinal hydroxyl groups on the β-glucan molecule in the presence of sodium periodate (NaIO_4_). Note that only β-(1→4)-linked units are oxidised.

Sodium periodate (NaIO_4_) oxidation was performed according to a method adapted from Coseri et al. (41). NaIO_4_ is known to selectively oxidise the two secondary, vicinal hydroxyl groups of the glucose monomers in polymers. This leads to the oxidative cleavage of the C2–C3 bond, which results in the opening of the glucopyranose ring and the formation of two aldehyde groups (Figure 2). To achieve maximal oxidation of all units that are potential substrates, namely all β-(1→4)-linked units that make up ∼70% of the polymer, 6 g oat or barley Chelex-treated extract was mixed in 0.7 L water with 1.5 g NaIO_4_/g β-glucan in the extract (1.6 eq. NaIO_4_ per β-(1→4)-linked glucose unit). The mixture was then gently stirred at RT for 24 h in the dark to prevent photo-oxidation (during which the extract fully dissolved), with the solution maintained at about pH 4 by means of 2 M NaOH and 0.5 M HCl solutions. The reaction was stopped by the addition of 5 mL glycerol and the oxidised β-glucan was precipitated with two volumes of ethanol. After 48 h at 4°C the samples were centrifuged (10 min, 3220 × *g*, 4°C), the collected solids freeze dried and milled by ball mill.

#### Acidic TEMPO Oxidation

**FIGURE 3:**
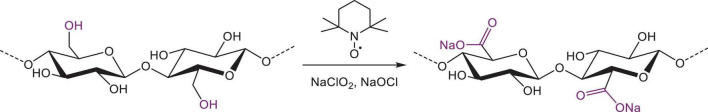
Oxidation of β-glucan in the presence of nitroxyl radical (TEMPO) and bleach to form a β-polyglucuronan.

An acidic TEMPO oxidation was performed on the samples according to the method by Tamura et al. (42), with some modifications. For maximal oxidation of all primary hydroxyl groups (Figure 3), a 0.2 M sodium acetate buffer (pH 4.7) with 60 mM NaClO_2_ and 6.1 mM TEMPO was prepared. While stirring, Chelex-treated oat or barley β-glucan extracts were added to a final concentration of 1% (w/v) (corresponding to roughly 60 mM primary hydroxyl groups). NaOCl was then added to a final concentration of 100 mM, the reaction vessels were capped and the samples were stirred in a water bath at 35°C for 24 h. The reaction was stopped by the addition of two volumes of ethanol and β-glucan was let to precipitate at 4°C for 48 h. Finally, the samples were centrifuged (10 min, 3220 × *g*, 4°C), freeze-dried and milled with a ball mill.

### *In vitro* Digestion and Determination of Dialysable Iron

The procedure used for the *in vitro* digestion was originally developed by Miller et al. (17) and adapted by Hurrell et al. (43). Additional slight adaptations were made in the present study to fit the experimental design. All β-glucan extracts, native and modified, were digested *in vitro* in triplicates. The digestion of the untreated and Chelex-treated samples was performed with and without a prior degradation of phytic acid in the samples by means of phytase. The following enzyme solutions were prepared freshly for each digestion: phytase solution (3 mg/mL = 30 U/mL), 3.2 g pepsin + 16.8 g 0.1 M HCl [resulting in 16% (w/w) pepsin], and both 0.4% (w/v) pancreatin and 2.5% (w/v) bile extract together in 0.1 M NaHCO_3_ (referred to as pancreatin/bile extract solution).

First of all, β-glucan solutions were prepared by mixing each extract with water [220 g solutions of 1% (w/w) actual β-glucan concentration, taking the β-glucan content into account], stirring the mixture for 30 min at RT and then shaking it vigorously in a dry incubator at 65°C for 2 h. For untreated oat, untreated barley, Chelex-treated oat and Chelex-treated barley β-glucan to achieve 1% actual β-glucan concentration, this corresponded to an extract concentration of 1.7, 2.2, 1.5, and 2.2%, respectively.

**FIGURE 4:**
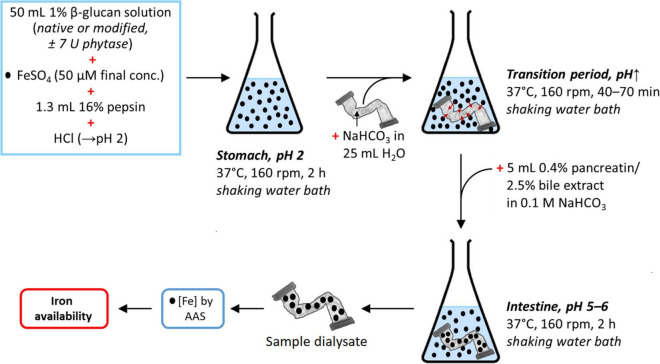
Schematic representation of the *in vitro* digestion simulation to determine the dialysability (and hence estimate availability) of iron in the presence or absence of native or modified β-glucan extracts, with or without prior phytase treatment. The references (± phytase) in this experiment were obtained analogously, but with 50 mL water instead of 1% β-glucan solution. The same is the case for the blanks (± phytase), where 50 mL water was used as in the references, but without added iron (•, Fe; see Figure 1 for a summary of all samples, references, and blanks).

The whole *in vitro* digestion is depicted in Figure 4. After cooling to RT and adjustment to pH 4–4.5 with 6 M HCl, either 1 mL water or 1 mL phytase solution (30 U; designated as “+P”) was added to the 1% β-glucan samples and references (220 mL water), which were then incubated overnight (16 h) in a shaking water bath at 40°C and 160 rpm. After cooling to RT, the solutions were adjusted to pH 2 using 6 M HCl in order to mimic gastric conditions. The solutions were then each divided into quadruplicate 50 mL aliquots (accomplished gravimetrically; density = 1.00 g/mL) in 100 mL Schott bottles. To each of these, 25 μL 0.1 M FeSO_4_ in 0.1 M HCl [50 μM final Fe(II)-concentration in each aliquot] and 1.3 mL 16% pepsin were added, followed by incubation for 2 h in a shaking water bath at 37°C and 160 rpm.

In the simulated transition from the stomach to the small intestine, a slow increase of pH is required. This is crucial in any attempt to simulate gastrointestinal digestion of food iron due to formation of ferrous and ferric hydroxide aggregates at elevated pH that eventually precipitate, lowering iron availability (17). The amount of base required to increase the pH of the different samples was determined by titration. Titratable acidity was determined on one of the 50 mL replicates by adding 5 mL of the pancreatin/bile extract solution followed by titration to pH 7.5 with 0.5 M KOH (roughly 1–3 mL of KOH solution required, depending on the sample). Titratable acidity was defined as the number of mmol KOH required to titrate the solution to pH 7.5. A segment of dialysis tubing 30 cm long (Spectra/Por Dialysis Membrane, MWCO 6–8 kDa, 32 mm flat width, Spectrum Labs, United States) containing 25 mL water and an amount of NaHCO_3_ equivalent to the titratable acidity measured previously (range of 0.5–1.5 mmol NaHCO_3_) was placed in each of the remaining three replicates, which were then incubated at 37°C in a shaking water bath (160 rpm) until the solution outside reaching pH ∼5, which took, depending on the sample, approximately 40–70 min. After that, 5 mL of the pancreatin/bile extract solution was added and the samples were incubated for an additional 2 h at 37°C in the shaking water bath (160 rpm). Finally, a 20 mL aliquot of the dialysate (sample inside the tube) was diluted two times and acidified with HCl to a final concentration of 0.1 M (pH 1) to keep the iron in solution. The samples were then further diluted according to their expected iron content to be below 10 μg/L (to be in the calibration range for analysis by AAS) and the iron content measured as described in section “Iron Content.”

In addition, a reference and a blank were also analysed: the reference was prepared and treated like the samples, but the β-glucan solution was replaced by water. In the blank, the β-glucan solution was also replaced by water, but no FeSO_4_ was added, in order to check possible contaminations of iron from the reagents and enzymes used for digestion (background iron levels). Indeed, iron was detected in low amounts also in the blank and blank+P, even though no FeSO_4_ and no β-glucan extract was added to these samples, with iron contaminations from the reagents (digestion enzymes and phytase) being the leading hypothesis. The iron levels were, however, >10-fold smaller than the observed Reference iron concentration, and hence low enough that it should not impact the analysis negatively. The results are reported after subtracting the blank background levels from the respective quantified iron concentrations of the samples and the reference (regular samples and reference minus blank; samples+P and reference+P minus blank+P), and given as iron availability relative to the regular reference in %. Cases were the measured iron concentration turned out to be slightly below the blank (and would hence give negative %-values) were not significantly different from the blank (*p* < 0.05) and are hence represented as 0% iron availability The phytase-treated samples and reference (reference+P and samples+P) were also expressed relative to the regular reference (without phytase) in %, in order to allow universal comparison of all samples.

### Statistical Analysis

Statistics of means and standard deviations (SDs) were calculated with Microsoft^®^ Excel 2016 (Microsoft Cooperation, United States). The iron concentrations in the dialysate of the samples, references, and blanks were compared using an independent sample *t*-test. Univariate analyses of variance (ANOVA) with additional *post hoc* testing including Tukey’s HSD were conducted to find out more about the effect of the different modifications and treatments on iron dialysability. For these tests, the Statistical Package for Social Science (IBM SPSS 22, Chicago, United States) was used. The threshold for indicating a significant difference was set at *p* < 0.05. Any error bars in figures correspond to SD.

## Results and Discussion

### Extract Composition

The composition of the β-glucan extracts was determined to check the efficiency of the extraction method and to be aware of possible influencing factors during the iron availability experiments (Table 1). The obtained total β-glucan contents were 58 and 46% (dwb) for oat and barley, respectively, and hence lower than the purities obtained by Bhatty (34). However, in the present study, due to the high quantity of extracts required for the experiments, the extraction method was simplified, and several purification steps were left out (mainly extensive dialysis and precipitation/re-dissolution steps), giving β-glucan contents that are more comparable to Zielke et al. (44). The Chelex-treatment led to a further purification of the oat β-glucan extract (+9 percentage points for OBG IF), conceivably due to the additional dissolution/ion-exchange/pH-change/precipitation steps, while surprisingly, for barley, the β-glucan content did not change.

**TABLE 1:** Total β-glucan, protein, phytic acid, and iron contents of the native oat (OBG) and barley (BBG) β-glucan extracts, as well as after Chelex-treatment to obtain the “iron-free” (IF) extracts.*^a^*

	β-Glucan (% dwb)	Protein (% dwb)	Phytic acid (% dwb)	Iron (μg/g)*^b^*	Fe:phytic acid (molar)
OBG	58 ± 1^b^	3.3 ± 0.1^b^	3.1 ± 0.5^a^	88.7 ± 1.9c	1:30
BBG	46 ± 0.1^a^	5.6 ± 0.0c	2.3 ± 0.4^a^	91.1 ± 1.4c	1:20
OBG IF	67 ± 0.2c	2.3 ± 0.0^a^	3.0 ± 0.5^a^	9.9 ± 2.1^a^	1:260
BBG IF	46 ± 2^a^	6.8 ± 0.1d	2.3 ± 0.7^a^	21.6 ± 0.4^b^	1:90

*^a^Values are means (±SD) of triplicate measurements. Different letters indicate significant differences (p < 0.05).*

*^b^Iron content reflects at the very least the accessible portion of intrinsic iron, and potentially not the actual total iron content due to the used method (see section “Iron Content”).*

The protein content of the barley β-glucan extract (5.6%) was higher than that of the oat extract (3.3%), which is in line with the results by Bhatty (34). Interestingly, the Chelex treatment led to a reduced protein content in the oat β-glucan extract (2.3%), but to an increased proportion of protein in the case of barley β-glucan (6.8%). Although the exact reasons are unknown for the different behaviour in extract composition changes after Chelex-treatment for oat vs. barley, they are most likely connected to differences in structure and composition of the native β-glucan extract starting materials.

The phytic acid contents were not significantly different between the two native extracts (*p* > 0.05), with a trend toward higher concentrations in the oat than in the barley β-glucan extract (3.1 and 2.3%, respectively). This trend correlates with the higher β-glucan content for the oat extract, and is in line with the used sources of whole grain flour for barley, and bran (where phytic acid is primarily located) for oat (45, 46). Comparing the results with literature values, phytic acid in the extracts is three to four times higher than in oat or barley flour (46). The Chelex treatment led to no change in phytic acid contents in the extracts (OBG/BBG IF) compared to the native materials (OBG/BBG). This is in sharp contrast to a recent study by Wang et al. (10) where an ion-exchange treatment of commercial oat β-glucan led to the concomitant removal of both iron and phytic acid.

The iron content of the untreated β-glucan extracts was similar for oat (88.7 ± 1.9 μg/g) and barley (91.1 ± 1.4 μg/g). The removal of the intrinsic iron using Chelex was comparable for oat and barley, with a reduction of 90 and 76%, respectively. With molar ratios of iron to phytic acid of at least 1:20 (BBG) and up to 1:260 (OBG IF), all intrinsic iron in the extracts can be assumed to be in the form of phytic acid chelates.

### Structural Characteristics

To characterise the β-glucan and investigate the effect of the modifications on the fibers, *M*_*w*_ and DP3/DP4 were determined before and after the different treatments by HPSEC and HPAEC-PAD, respectively (Table 2). Literature values for the *M*_*w*_ of cereal β-glucans are very variable because of differences in extraction and *M*_*w*_ determination methods (47). However, in this study, there was a significant difference in the *M*_*w*_ of unmodified oat and barley β-glucan (*p* < 0.05), with oat β-glucan being bigger (957 kDa) than barley β-glucan (644 kDa), which is in agreement with the literature conducted under comparable conditions (27, 48).

**TABLE 2:** Weight-average molecular weights (*M*_*w*_) and molar DP3/DP4 ratios determined by means of HPSEC and lichenase treatment/HPAEC-PAD, respectively.

β-Glucan extract	*M*_*w*_ (kg/mol)*^a^*	DP3/DP4*^a^*
OBG	957 ± 37 d	1.50 ± 0.09 a
BBG	644 ± 16 c,d	2.20 ± 0.14 c
OBG IF	567 ± 38 c	1.44 ± 0.04 a
BBG IF	662 ± 28 d	2.05 ± 0.10 b,c
OBG HCl	74 ± 20 a	1.40 ± 0.13 a
BBG HCl	62 ± 8 a	1.84 ± 0.12 b
OBG TEMPO	442 ± 7 b	–*^c^*
BBG TEMPO	529 ± 46 c	–*^c^*
OBG NaIO_4_	∼5*^b^*	–*^c^*
BBG NaIO_4_	∼5*^b^*	–*^c^*

*^a^Values are means (±SD) of triplicate measurements. Different letters indicate significant differences (p < 0.05).*

*^b^Molecular weight reported as peak molecular weight (M_p_), determined from the peak retention time and the SEC calibration curve, as the sample signal was partially merging with the solvent peak in the SEC-analysis, and did not allow determination of the exact weight-average molecular weight.*

*^c^No significant levels of DP3 and DP4 oligomers were detected after lichenase treatment of the oxidised samples (TEMPO or NaIO_4_) due to the extensive oxidation of the polysaccharide.*

Results show that Chelex treatment (“iron-free” samples; OBG/BBF IF) reduced the *M*_*w*_ of oat β-glucan (−41%; *p* < 0.001) but not that of barley β-glucan (*p* > 0.05). This could be due to the slightly different composition of the two extracts, making them to a different degree susceptible to degradation. For example, the liberation of iron during the Chelex treatment might allow for the Fenton reaction to occur, with hydroxyl radicals (HO) inducing small amounts of cleavage observed on the oat β-glucan backbone. With a slightly larger proportion of iron liberated in the Chelex treatment for the OBG extract (90 vs. 76% for BBG extract), more free iron was available to catalyse the Fenton reaction, and with HO indiscriminately attacking organic substrates, the purer oat extract (58% β-glucan content vs. 46% for BBG; Table 1) would be affected as observed the most. The produced Chelex treated, “iron-free” (IF) materials were used to produce modified samples. Acid hydrolysis, for example, led as expected to a relatively high degree of chain scission and significantly reduced the molecular size by roughly 8 times for oat and 11 times for barley (both *p* < 0.001). This treatment was used to study the effect of the reduction of *M*_*w*_ on the iron-binding by cereal β-glucan without any other alteration of the structure.

2,2,6,6-Tetramethylpiperidine 1-oxyl treatment reduced the *M*_*w*_ by only ∼20% for both oat and barley compared to the used OBG/BBG IF starting material (*p* < 0.001 for both). TEMPO oxidation was chosen because it is a reasonably mild modification that allows to investigate the effect of oxidation on iron-binding by β-glucan without dramatically reducing the *M*_*w*_ of the fiber, while introducing potential new binding sites for iron, namely carboxylate groups. It is interesting, however, that the *M*_*w*_ reduction was considerably lower compared to our previous study, where TEMPO oxidation decreased *M*_*w*_ by a factor of 6 for both oat and barley β-glucan, although the starting *M*_*w*_ was higher (1600 and 1300 kg/mol for oat and barley β-glucan, respectively) (33).

As previously observed, NaIO_4_ oxidation of β-glucan led to drastically reduced *M*_*w*_ (33) presumably due to susceptibility to β-elimination induced by the newly introduced carbonyl groups, leading to backbone cleavage (49). In the literature, extensive degradation was for example also observed for alginate upon treatment with sodium periodate, especially when complete oxidation was attempted (50).

The molar DP3/DP4 ratio, which represents the fine structure of the fiber and is known to influence certain properties (solubility, gelation rate), was determined to characterise more thoroughly the extracted β-glucan (Table 2). DP3/DP4 for the untreated oat and barley β-glucan extracts were with 1.5 and 2.2, respectively, in the range of reported literature values (28, 51, 52). As for the treatments and modifications, both Chelex-treatment (OBG/BBG IF) and acid hydrolysis (OBG/BBG HCl) did not significantly change the DP3/DP4 ratio for each step (*p* > 0.05). However, virtually no DP3 and DP4 oligomers were detected after lichenase treatment of the oxidised samples (TEMPO or NaIO_4_), confirming an extensive oxidation as previously observed (33).

### *In vitro* Digestion and Dialysis: Estimation of Iron Availability

After the *in vitro* digestion, the iron concentrations in the dialysis tubes were determined by AAS and blank-corrected. Of the added iron(II) in the reference sample (without β-glucan), two thirds was recovered of the calculated theoretical equilibrium concentration in the final total volume, but the difference between calculated (30.7 μM Fe) and observed blank-corrected concentration (20.6 ± 4.5 μM) was not significant (*p* > 0.05). Such a moderate, statistically insignificant loss of one third indicates that most of the iron(II) was successfully kept in solution, despite its propensity to easily oxidise in pure aqueous solutions in the presence of dissolved oxygen and precipitate as Fe(III) hydroxides at pH > 4 (53). Both the decent iron recovery and its relatively large SD can be explained by a well working method to keep iron in solution that is, however, not perfect: (1) the slow pH transition from the simulated stomach (pH 2) to the conditions in the small intestine (pH 5–6) with titrimetrically determined exact NaHCO_3_ amounts that were added as solution in dialysis tubes according to the method developed by Miller et al. (17) and adapted by Hurrell et al. (43) largely, but not fully, prevented iron hydroxide precipitation, and (2) the known stabilising effect of bile acids (from the added bile extract) helped in keeping ferrous iron more or less dissolved (54, 55).

**FIGURE 5:**
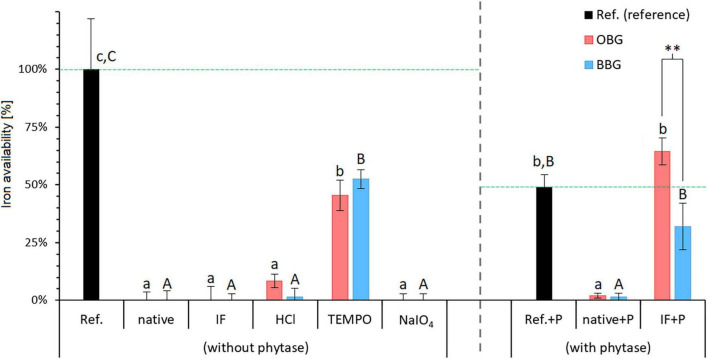
Iron availability in the presence of various dissolved native or modified β-glucan extracts from oat (OBG) and barley (BBG) obtained from the *in vitro* digestion study. The available iron as measured by the amount that crossed the dialysis membrane from the digestion mixture is presented as percentages relative to the reference (Ref.; no β-glucan added). Horizontal dashed lines in green emphasise the 100% reference levels for regular (without phytase) and phytase-treated samples (with phytase). Note that for the samples with phytase (+P), the reference (Ref.+P) exhibits a substantially lower available iron content of 49% compared to the regular reference [Ref. (without phytase) = 100%; corresponding to a blank-corrected concentration of 20.6 μM iron in the dialysate]. Different lowercase and uppercase letters indicate statistically significant differences within oat and barley samples, respectively (*p* < 0.05). Differences between oat and barley samples of the respective type are indicated by asterisks (^**^, *p* ≤ 0.01).

Figure 5 shows the iron availability of all samples expressed in percent, relative to the experimental reference sample (Ref.). All structurally unmodified samples (OBG, BBG, OBG IF, BBG IF) exhibited no available iron, presumably due to their large phytic acid contents (2.3–3.1%; Table 1) that represent a 9- to 13-fold molar excess compared to the total iron during the *in vitro* digestion (both intrinsic and extrinsic iron; see Fe_(total)_:phytic acid in Table 3). In contrast to free Fe^2+^ and the presumed Fe(II) complexes of bile acids and their conjugates, which diffuse into the dialysis tubing (as seen in the reference sample without added β-glucan extract), iron-phytate complexes seem to be not free to travel through the membrane, but are somehow immobilised. This could take place by either a strong interaction of the iron-phytate complex (net negative charge) with residual cereal proteins (with local positive charges) that are too large to pass the dialysis membrane (MWCO of 6–8 kDa), or simply due to the poor solubility of the phytate chelates.

**TABLE 3:** Calculated concentrations of contaminants from the β-glucan extract material in the prepared 1% β-glucan solution used for the *in vitro* digestion.*^a^*

	Phytic acid (μM)	Protein (g/L)	Total Fe (μM)*^*b*^*	Intrinsic Fe relative to total Fe (%)	Fe_(total)_:phytic acid (molar ratio)
OBG	820 ± 140 a	0.57 ± 0.02 b	78 ± 1 c	36	1:11
BBG	750 ± 120 a	1.22 ± 0.01 c	86 ± 1 d	42	1:9
OBG IF	680 ± 120 a	0.34 ± 0.01 a	53 ± 1 a	5	1:13
BBG IF	740 ± 230 a	1.47 ± 0.02 d	58 ± 1 b	14	1:13

*^a^Phytic acid and protein are given as intrinsic concentrations in the 50 mL solution, while intrinsic iron (Fe) is given relative to the total iron content. Different letters indicate significant differences (p < 0.05).*

*^b^Absolute total iron concentration = 50 μM extrinsic iron + intrinsic iron from the extract material.*

#### Phytase Treatment

Interestingly, a comparison of the two references with and without phytase (Ref. and Ref.+P) shows that the dialysate of Ref.+P contains only half of the amount of iron compared to the untreated Reference (49 vs. 100%, respectively; *p* < 0.001). As phytase itself is not known to bind iron to an appreciable amount, an inactive component of the commercial enzyme product is more likely to be responsible for the observed lowered iron availability. For example, even an inconspicuous protein such as bovine serum albumin that is often added in enzyme preparations is known to bind iron in the presence of hydrogen carbonate ions (56). Therefore, iron-binding to a component of the commercial phytase material is a reasonable explanation (7). However, regardless of the cause for the lower Ref.+P iron availability, since phytase-specific blank+P and Ref.+P are used, comparisons of the phytase-treated sample results can still give useful information.

Phytase treatment for the native materials, namely OBG/BBG native+P, made no significant difference in iron availability (*p* > 0.05), indicating that, despite of the phytase treatment that should hydrolyse phytic acid, virtually all iron is still bound (∼2% relative iron availability). If a Chelex treatment is applied before the phytase, however, the retention of iron is as expected diminished, with the dialysate of both OBG IF+P (65 ± 6%) and BBG IF+P (32 ± 10%) exhibiting iron availabilities that are not significantly different from the phytase reference Ref.+P (49 ± 6%; *p* > 0.05; all values shown relative to the normal Ref.). The fact that in the Chelex-treated, iron-free samples plus phytase (IF+P) the dephosphorylation of phytic acid strongly increased the amount of available iron for both oat and barley (*p* = 0.007 and *p* < 0.001, respectively) is in line with what was suggested by Hurrell (8), who states that complete degradation of phytic acid would improve iron bioavailability perhaps fivefold or more and that more modest reductions in phytic acid content may not significantly improve iron absorption. The stronger effect of the phytase treatment on the Chelex-treated samples (IF+P) compared to the untreated ones (native+P) might be explained with the difference in intrinsic iron content between the two types of samples (Table 1). The untreated extracts in fact, have a higher intrinsic iron content than the Chelex-treated ones, in which the iron content was reduced by 90% for oat and 76% for barley. It can be hypothesised that this iron (and other mineral cations such as calcium and magnesium) are bound to phytic acid molecules as insoluble phytate complexes, providing a (steric) hindrance to phytase and rendering its action less effective.

#### Differences Between Oat and Barley β-Glucan

The only significant difference of iron-binding between an oat and barley sample pairs was observed for OBG IF+P vs. BBG IF+P (*p* = 0.0084; Figure 5). This might arise in part from a non-complete enzymatic hydrolysis of phytic acid in the barley sample due to the slightly less successful removal of iron (and presumably other metal cations as well) during the Chelex treatment compared to the oat sample (Table 1), and hence small residual phytic acid amounts due to the hampered accessibility of phytase described above. However, assuming full degradation of phytic acid for both oat and barley IF+P samples, which is also conceivable due to the statistically indifferent phytic acid concentration in 1% β-glucan solution (680–740 μM; *p* > 0.05), the observed discrepancy in iron availability could also in part reflect the large differences of protein concentrations in the digest solution due to the different extract compositions and amounts needed to reach 1% β-glucan (Table 3). The fourfold higher protein content in the barley solutions (1.47 g/L for BBG IF vs. 0.34 g/L for OBG IF; *p* < 0.001) can explain the lower trend in available iron for these samples: once the phytic acid is digested, a small binding effect exerted by the intrinsic proteins is unveiled, which confirms phytic acid as the major player in binding iron for β-glucan extracts, but reveals proteins to play an additional minor role, as peptides containing serine phosphate or high proportions of carboxylic acid groups are known to interact with iron (7).

#### Effect of Modifications

**FIGURE 6:**
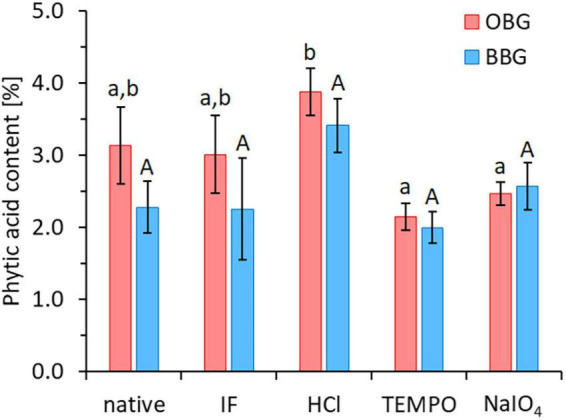
Phytic acid contents of the native and Chelex-treated (“iron-free”, IF) oat (OBG) and barley (BBG) β-glucan extracts, as well as the three modifications (HCl, TEMPO, NaIO_4_) that used OBG/BBG IF as starting material. Different lowercase and uppercase letters indicate statistically significant differences within oat and barley samples, respectively (*p* < 0.05). There were no significant differences between oat and barley pairs for each treatment type (*p* > 0.05).

The impact of the different modifications (TEMPO oxidation, NaIO_4_ oxidation, and acid hydrolysis) on iron availability was also investigated (Figure 5). While TEMPO oxidation significantly increased the availability of iron for oat and barley β-glucan extracts (*p* < 0.001 for both), in the case of NaIO_4_ oxidation and acid hydrolysis, no significant change in iron availability compared to their starting materials (OBG/BBG IF) was observed (*p* > 0.05). On the other hand, none of these modifications lead to significant changes (*p* > 0.05) in phytic acid contents compared to their starting material OBG IF/BBG IF (range of 2.0–3.9%; Figure 6). The roughly constant phytic acid levels were expected, since phytic acid has neither free vicinal diols as substrate for NaIO_4_ nor primary alcohol groups for TEMPO, and the acid hydrolysis (0.1 M HCl, 50°C, 24 h) was too mild to affect it, as phytic acid needs very harsh conditions to be hydrolysed quantitatively [2 M HCl for 24 h at 120°C; (57)]. Nevertheless, while the treatments did not change the measured total phosphate content from phosphorylated inositols (IP*_*n*_*), with six phosphate esters on the inositol core corresponding to phytic acid (IP_6_), the IP*_*n*_* profiles might be different for the different β-glucan materials. The iron-binding capacity of IP*_*n*_* is the highest for *n* = 6 (phytic acid) and decreases with decreasing *n*, with *n* < 3 being necessary to improve iron absorption from cereals and legumes (58, 59). Due to the large molar excess of phytic acid from the extracts compared to the total iron in the model system, >90% of the phytic acid (IP_6_) would need to be degraded to IP_1_ and IP_2_ to expect a dramatic increase in iron availability, which would also reflect in the total phytic acid content measured with the used commercial kit as a decrease to less than 1/3 of the original content. However, such a substantial decrease in phytic acid content was not observed for any of the modifications. While the constant phytic acid levels explain the constant diminished iron availability for the HCl and NaIO_4_ samples, other effects must be at play for the TEMPO materials.

TEMPO oxidation of Chelex-treated (IF) β-glucan, producing a β-glucuronan, led to an increase from 0% to around 50% relative iron availability for both oat and barley extract (*p* < 0.001; Figure 5), with no change in phytic acid levels that could explain this change in behaviour (Figure 6). However, the mineral bioavailability in phytic acid-containing food and the solubility of phytic acid and its phytate salts are a complex equilibrium that depends on, among others, the pH, the metal cation (individual or mixtures), its concentration and molar ratio to phytic acid, as well as on the presence of competing chelating agents (60, 61). For instance, in a previous study, residual, intrinsic phytic acid and iron was successfully removed from a commercial high viscosity oat β-glucan material (>95% purity) by extensive dialysis of a 1% solution against 1 mM EDTA and water, followed by precipitation with ethanol and freeze-drying (62). The successful removal of phytic acid/iron (and presumably also Ca^2+^, Mg^2+^…) was accompanied by a clearing of the 1% β-glucan solution from slightly opaque (presumably due to insoluble, finely dispersed phytate/protein complexes) to virtually transparent, thanks to the solubilising effect of EDTA on phytate-metal cation complexes.

**FIGURE 7:**
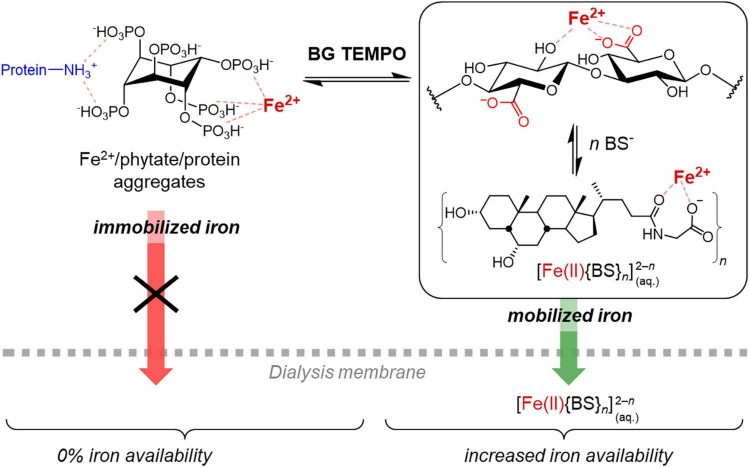
Schematic representation of the proposed mechanism to explain the observed effect of the TEMPO-oxidised β-glucan material (BG TEMPO) with increased iron availability despite substantial phytic acid contents, by transferring Fe^2+^ from large iron/phytate/protein aggregates to soluble iron/bile salt (BS) complexes that are able to cross the dialysis membrane. Glycohyodeoxycholate is shown as BS representative of the used bile extract.

Similarly, the new carboxylic acid groups introduced in the TEMPO-treated polysaccharide of this study may compete with phytic acid for the added iron during the *in vitro* digestion model (and other minerals such as Mg^2+^ and Ca^2+^), creating a dynamic situation by breaking up phytate aggregates due to the removal of residual metal cations from the phytate complexes (Figure 7). Glucuronates, the monomer units of the oxidised β-glucan, are known to bind divalent metal cations such as Ca^2+^, Mg^2+^, and Zn^2+^ (63). Once bile extract is added (after the pH transition), other carboxylic acids, namely bile salts (BSs), are present that may compete with the polyglucuronate–metal cation complexes. β-Polyglucuronates are known to lack the high stereo-specificity and the well-defined chelation sites, e.g., characteristic for α-(1→4)-polygalacturonan, and instead present a “cloud” of favourable positions for the cation along the chain that allows for a more dynamic mobilisation of metal cations rather than a tight, strong binding (64). Hence, the moderate strength, dynamic iron-binding of polyglucoronates OBG/BBG TEMPO allow them to act as intermediary Fe^2+^ transporters from immobilised Fe^2+^/phytate/protein aggregates to [Fe(II){BS}*_*n*_*]^2–^*^n^* complexes small enough to pass through the dialysis membrane (Figure 7), leading to an equilibration of iron concentration between outside and inside the dialysis tubing, at least to a relative iron availability of roughly 50%. Therefore, instead of producing large, strong aggregates of phytic acid, iron and extract constituents that prevent the iron from crossing the dialysis membrane (as was the case for native and IF β-glucan extracts), the interaction with TEMPO-treated β-glucan might be more dynamic, with the oxidised polysaccharide facilitating to keep iron in solution, but not holding on it strong enough to prevent it from crossing the membrane as smaller BS complex.

Although there is an abundance of literature about iron bioavailability in foods, only few studies investigate or discuss the role of the partly soluble dietary fibers such as β-glucan (24, 25, 40, 65). However, it is nowadays an established fact that phytic acid plays a major role in the observed reduced bioavailability of the iron present in cereals and other vegetable food, as also confirmed by Wang et al. (10, 11). As previously discussed, both oat and barley β-glucan extracts contain high levels of phytic acid (Table 2) and it is well known that even small amounts of phytic acid can bind considerable amounts of iron (9, 46). Therefore, the key role of phytic acid in iron-binding is once more confirmed by the present study. While the true iron bioavailability depends on a complex combination of factors, the results of this study offer a relative comparison, which can help to understand how processing of oat and barley β-glucan materials affects the retention of iron, and, as a consequence, iron availability.

## Conclusion

Oat and barley β-glucan extracts, native or modified (TEMPO oxidation, sodium periodate oxidation and acid hydrolysis), were studied to investigate the impact of molecular structure and phytic acid content on iron availability. To do this, an *in vitro* digestion featuring a reverse dialysis system was performed to mimic the behaviour of the fiber extracts in the human gastro-intestinal tract in the presence of iron. The results confirm that the β-glucan polysaccharide itself does not directly impede iron availability under the digestion model conditions. On the other hand, they confirm the major impact phytic acid has on the inhibition of iron absorption and suggest that associated protein may also play a part in iron-binding once phytic acid effects are eliminated.

In contrast to NaIO_4_ oxidation or partial hydrolysis by HCl, TEMPO oxidation was an effective modification for substantially improving iron availability for both oat and barley samples despite unchanged phytic acid levels, confirming our hypothesis that structural modification of β-glucan changes iron availability of its extracts for the latter, while refuting it for the other two. Phytase treatment was only successful in liberating iron to increase its availability for Chelex-treated samples, but not native extract, presumably due to the reduced amounts of minerals that complex phytic acid in the former, which has major implications when attempting to increase iron bioavailability through phytase treatments in β-glucan containing food products. Due to the large molar excess of phytic acid in cereals, its degradation through phytase must be close to quantitative to markedly increase iron availability. Fine structure differences between oat and barley β-glucan might hence play an indirect role by limiting access of phytase through physical entrapment due to differences in gelation. Additional *in vivo* and *in vitro* experiments studying the interactions between β-glucan, phytic acid, phytase, and iron are required to further investigate these mechanisms and ensure optimal iron bioavailability in foods.

## Data Availability Statement

The original contributions presented in this study are included in the article/supplementary material, further inquiries can be directed to the corresponding author.

## Author Contributions

EM: investigation, methodology, writing – original draft, and visualisation. OZ-W: investigation and writing – original draft. SB: investigation, writing – review and editing, visualisation, and formal analysis. LN: conceptualisation, funding acquisition, supervision, and resources. All authors contributed to the article and approved the submitted version.

## Conflict of Interest

The authors declare that the research was conducted in the absence of any commercial or financial relationships that could be construed as a potential conflict of interest.

## Publisher’s Note

All claims expressed in this article are solely those of the authors and do not necessarily represent those of their affiliated organizations, or those of the publisher, the editors and the reviewers. Any product that may be evaluated in this article, or claim that may be made by its manufacturer, is not guaranteed or endorsed by the publisher.
